# Association of Electronic Cigarette Regulations With Electronic Cigarette Use Among Adults in the United States

**DOI:** 10.1001/jamanetworkopen.2019.20255

**Published:** 2020-01-31

**Authors:** Yang Du, Buyun Liu, Guifeng Xu, Shuang Rong, Yangbo Sun, Yuxiao Wu, Linda G. Snetselaar, Robert B. Wallace, Wei Bao

**Affiliations:** 1 Department of Epidemiology, University of Iowa College of Public Health, Iowa City

## Abstract

**Importance:**

Millions of Americans use electronic cigarettes (e-cigarettes). A growing number of state and local governments have started to draft and implement laws regarding the sale, marketing, and use of e-cigarettes. The association of US state regulations regarding e-cigarettes with e-cigarette use remains unknown.

**Objective:**

To examine the association of US state regulations regarding e-cigarettes with current e-cigarette use among adults in the United States.

**Design, Setting, and Participants:**

This cross-sectional study included adults aged 18 years or older from the 2016 and 2017 Behavioral Risk Factor Surveillance System, which is a nationwide, telephone-administered survey that collects state-representative data on health-related risk behaviors, chronic health conditions, and use of preventive services. Data analysis was performed from February 1, 2019, to April 31, 2019.

**Exposures:**

United States state laws regulating e-cigarette use, including prohibiting e-cigarette use in indoor areas of private workplaces, restaurants, and bars; requiring retailers to purchase a license to sell e-cigarettes; prohibiting self-service displays of e-cigarettes; prohibiting sales of tobacco products, including e-cigarettes, to persons younger than 21 years; and e-cigarette taxes.

**Main Outcomes and Measures:**

Current use of e-cigarettes.

**Results:**

Among 894 997 participants aged 18 years or older (503 688 women [51.3%], 679 443 non-Hispanic white [62.6%], 71 730 non-Hispanic black [16.3%], 69 823 Hispanic [11.4%], and 74 001 non-Hispanic other races [9.8%]), 28 907 (weighted prevalence, 4.4%) were currently using e-cigarettes. The age-standardized weighted prevalence of current e-cigarette use varied across US states and territories, from 1.0% in Puerto Rico to 6.2% in Guam. After adjustment for demographic, socioeconomic, and lifestyle factors, including conventional cigarette use, the odds ratios of current e-cigarette use were 0.90 (95% CI, 0.83-0.98) for state laws prohibiting e-cigarette use in indoor areas of private workplaces, restaurants, and bars; 0.90 (95% CI, 0.85-0.95) for state laws requiring retailers to purchase a license to sell e-cigarettes; 1.04 (95% CI, 0.99-1.09) for state laws prohibiting self-service displays of e-cigarettes; 0.86 (95% CI, 0.74-0.99) for state laws prohibiting sales of tobacco products, including e-cigarettes, to persons younger than 21 years; and 0.89 (95% CI, 0.83-0.96) for state laws applying taxes to e-cigarettes.

**Conclusions and Relevance:**

These findings suggest that several state regulations regarding e-cigarettes may be associated with reduced e-cigarette use among US adults.

## Key Points

**Question:**

Are US state regulations regarding electronic cigarettes (e-cigarettes) associated with current e-cigarette use among US adults?

**Findings:**

This cross-sectional study used national data from 894 997 participants aged 18 years or older from the 2016 and 2017 Behavioral Risk Factor Surveillance System surveys. State regulations prohibiting e-cigarette use in indoor areas, requiring retailers to purchase a license to sell e-cigarettes, prohibiting sales of tobacco products to persons younger than 21 years, and applying taxes to e-cigarettes were associated with a lower prevalence of current e-cigarette use among US adults.

**Meaning:**

Several state regulations regarding e-cigarettes may be associated with reduced current e-cigarette use among US adults.

## Teaser

This cross-sectional study examines the association of US state regulations regarding electronic cigarettes with current electronic cigarette use among adults in the United States.

## Introduction

Electronic cigarettes (e-cigarettes) have been marketed as a safer alternative to conventional cigarettes and an adjunct for smoking cessation.^1,2,3^ Millions of US individuals use e-cigarettes.^4^Moreover, although the prevalence of conventional cigarette smoking is declining,^5^ e-cigarette use has become more popular in the US population.^6,7^ In the United States, 3.2% of adults currently used e-cigarettes in 2018, and the rate was even higher among young adults aged 18 to 24 years, with a current use rate of 7.6%.^8^ Thus far, evidence regarding the efficacy and safety of e-cigarettes for successful smoking cessation is controversial and inconclusive.^2,9,10,11,12,13,14,15,16^ A limited number of clinical trials^13,14,16^ on the use of e-cigarettes for smoking cessation have been published, and the results are conflicting. Moreover, initial use of e-cigarettes may increase the risk of subsequent conventional cigarette smoking among youth and young adults.^17^ In addition, although e-cigarettes generally contain fewer numbers and lower levels of harmful substances (eg, nicotine) than combustible tobacco cigarettes,^18,19,20^ most e-cigarettes contain and emit numerous other substances that are potentially toxic, such as heavy metals, propylene glycol, volatile organic chemicals, and carcinogenic agents.^21,22^ More than 7000 flavoring chemicals, many with uncertain toxicity, are currently marketed for e-cigarettes.^23^ Recent studies have shown that the use of e-cigarettes is associated with endothelial cell dysfunction, oxidative stress, impaired vascular function,^24,25,26^ increased risk of cardiovascular diseases,^27,28,29^ and other long-term adverse health outcomes.^30^

In May 2016, the US Food and Drug Administration released a rule^31^ to regulate the manufacturing, distribution, and marketing of e-cigarettes as a tobacco product. In addition, state, local, tribal, and territorial governments maintain broad regulatory authority and have taken additional, more-stringent actions.^32^ Since 2010, a growing number of states and local governments have started to draft and implement laws regarding the sale, marketing, and use of e-cigarettes.^33^ Currently, e-cigarette–related regulations and policies implemented by some US states include laws prohibiting e-cigarette use in indoor areas of private workplaces, restaurants, and bars; laws requiring retailers to purchase a license to sell e-cigarettes; laws prohibiting self-service displays of e-cigarettes; laws prohibiting sales of tobacco products, including e-cigarettes, to persons younger than 21 years; and laws applying taxes to e-cigarettes.^33^ Some states have adopted multiple laws, whereas others have not.^32,33^ Previous studies^4,34,35^ have found substantial variations in the prevalence of current e-cigarette use among adults across US states; however, the association of state laws regarding e-cigarettes with e-cigarette use is still unknown.

In this cross-sectional study using data from a large nationwide surveillance program covering all 50 states and participating territories in the United States, we examined the association of US state regulations regarding e-cigarettes with current e-cigarette use among adults in the United States. We hypothesized that state laws regarding e-cigarettes may be associated with lower prevalence of e-cigarette use among adults across US states.

## Methods

### Study Population

The Behavioral Risk Factor Surveillance System (BRFSS) is the premier system of health-related telephone surveys in the United States. The BRFSS collects data from US residents in all 50 states, the District of Columbia, and 3 US territories (Puerto Rico, Guam, and US Virgin Islands) regarding their health-related risk behaviors, chronic health conditions, and use of preventive services. The BRFSS completes more than 400 000 adult interviews each year, making it the largest continuously conducted health survey system in the world. With technical and methodological assistance from the Centers for Disease Control and Prevention (CDC), state health departments use in-house interviewers or contract with telephone call centers or universities to administer the BRFSS surveys continuously through the year. In the BRFSS, a sample record is 1 telephone number in the list of all telephone numbers the system randomly selects for dialing. The survey is conducted using random digit dialing techniques on both landlines and cellular telephones. Each state samples from adults living in private residences via landline and cellular telephone. A disproportionate stratified sampling design is usually used in the landline sample, which requires interviewers to collect information on the number of adults living within a residence and then select randomly from all eligible adults. Cellular telephone sampling frames are commercially available, and the system can call random samples of cellular telephone numbers, according to specific protocols. A detailed description of the BRFSS survey design, questionnaires, and data collection can be found on the BRFSS website.^36^

The BRFSS is unique because it is designed to be state representative, which allows estimation of state-level e-cigarette use among the general population in the United States. We used data from the BRFSS 2016 and 2017 surveys, because the BRFSS first started to collect information on e-cigarette use in 2016. During 2016, all 50 states, the District of Columbia, the Commonwealth of Puerto Rico, Guam, and US Virgin Islands collected BRFSS data, whereas during 2017, US Virgin Islands did not collect BRFSS data; thus, we did not include US Virgin Islands in the present analysis. The term “state” is used to refer to all areas participating in the BRFSS, including the District of Columbia, Guam, and the Commonwealth of Puerto Rico.

The University of Iowa institutional review board determined that the present study is exempt from the need to obtain informed consent because of the use of deidentified data. This study follows the Strengthening the Reporting of Observational Studies in Epidemiology (STROBE) reporting guidelines.^37^

### Assessment of e-Cigarette Use

Participants were asked about their history of cigarette smoking and e-cigarette use. For e-cigarettes, the BRFSS questionnaire included a brief introduction, as follows: “Electronic cigarettes (e-cigarettes) and other electronic ‘vaping’ products include electronic hookahs (e-hookahs), vape pens, e-cigars, and others. These products are battery-powered and usually contain nicotine and flavors such as fruit, mint, or candy.” Participants were first asked, “Have you ever used an e-cigarette or other electronic ‘vaping’ product, even just one time, in your entire life?” Those who responded yes were further asked, “Do you now use e-cigarettes or other electronic ‘vaping’ products every day, some days, or not at all?” Similar to previous studies,^4,7^ we considered participants who answered yes to the first question and then responded either “every day” or “some days” to the second question as current users of e-cigarettes. In this study, we focused on current use of e-cigarettes.

### Ascertainment of State Laws Regarding e-Cigarettes

For each type of state law, we considered participants as being exposed to the state law if they were living in the state where the state law regarding e-cigarette use was implemented no later than January 1 of the survey year. Information about whether and when state laws regarding e-cigarettes were implemented was derived for all the 50 states, the District of Columbia, Puerto Rico, and Guam according to the CDC State Tobacco Activities Tracking and Evaluation System.^33^ Consistent with a recent report by the CDC,^33^ US states were classified according to whether the state has implemented each type of the following state laws: prohibiting e-cigarette use in indoor areas of private workplaces, restaurants, and bars; requiring retailers to purchase a license to sell e-cigarettes; prohibiting self-service displays of e-cigarettes; prohibiting sales of tobacco products, including e-cigarettes, to persons younger than 21 years; and e-cigarette taxes. Because we included 2 years of data from BRFSS, we linked state laws to each participant according to the year the survey was completed. For example, participants in the 2016 survey were regarded as being exposed to the laws if the states where they were living implemented the specific laws no later than January 1, 2016. Similarly, for participants in the 2017 survey, if the states where they were living implemented the specific laws no later than January 1, 2017, they were regarded as being exposed to the laws.

### Assessment of Covariates

Information on participants’ age, gender, race/ethnicity, education, family income, cigarette smoking, alcohol drinking, and physical activity was collected during the interview. Race/ethnicity was categorized as non-Hispanic white, non-Hispanic black, Hispanic (Mexican and non-Mexican Hispanic), and other race/ethnicity. Education was grouped as less than high school, high school, attended college, or graduated from college. Annual family income was categorized as less than $15 000, $15 000 to less than $25 000, $25 000 to less than $35 000, $35 000 to less than $50 000, and $50 000 or more. Current alcohol intake was categorized as none (0 drinks per week), moderate drinking (1-14 drinks per week for men and 1-7 drinks per week for women), and heavy drinking (>14 drinks per week for men and >7 drinks per week for women). Participants were categorized as nonsmokers, former smokers, current smokers some days, and current smokers every day. Physical activity was categorized according to whether the participant had engaged in leisure time physical activity during the past 30 days other than at their regular job.

### Statistical Analysis

We incorporated survey sampling weights, provided by the CDC along with the BRFSS data set, in all analyses to account for the complex survey design and to make the findings generalizable to the general population at both the state and national level. Since 2011, the BRFSS has used a technique called “raking” to weight BRFSS survey data to account for known proportions of age, race/ethnicity, gender, geographic region, and other socioeconomic characteristics such as education level and home ownership status of participants. A detailed description of the weighting processes in BRFSS has been published elsewhere.^38^

Characteristics of the study participants are presented as means (standard errors) for continuous variables or counts (percentage) for categorical variables. Generalized linear models were used to compare differences in continuous variables, and χ^2^ tests were used for categorical variables. We calculated the age-standardized prevalence of current e-cigarette use according to 2010 US Census population data using age categories of 18 to 44 years, 45 to 64 years, and 65 years or older. We calculated the difference in prevalence of e-cigarette use in each state between 2016 and 2017. We used multivariable logistic regression models to estimate odds ratios and 95% CIs of current e-cigarette use associated with state laws regarding e-cigarettes. Model 1 was adjusted for age and gender. Model 2 was further adjusted for race/ethnicity, education, family income, smoking status, alcohol intake, and physical activity. We conducted stratified analyses according to age (18-24, 25-44, 45-64, and ≥65 years), gender (male vs female), race/ethnicity (white vs nonwhite), education level (high school or less vs more than high school), annual family income (<$50 000 vs ≥$50 000), and smoking status (current smoker every day, current smoker some days, former smoker, or never smoker). Interaction analyses were performed by including multiplicative terms of each exposure variable with effect modifier variable in the aforementioned multivariable models.

All analyses were performed using survey procedures in SAS statistical software version 9.4 (SAS Institute). Two-sided *P* < .05 was considered statistically significant. Data analysis was performed from February 1, 2019, to April 31, 2019.

## Results

In this study, we included 894 997 adults aged 18 years or older with data on state residence and e-cigarette use available (503 688 women [51.3%], 679 443 non-Hispanic white [62.6%], 71 730 non-Hispanic black [16.3%], 69 823 Hispanic [11.4%], and 74 001 non-Hispanic other races [9.8%]); 28 907 participants (weighted prevalence, 4.4%) were currently using e-cigarettes. Electronic cigarette users were more likely than nonusers to be male (15 068 [60.1%] vs 375 940 [48.1%]; difference, 12.0%; 95% CI, 11.4%-12.6%; *P* < .001), non-Hispanic white (22 174 [70.6%] vs 657 269 [62.2%]; difference, 8.4%; 95% CI, 7.8%-9.0%; *P* < .001), current smokers (15 982 [51.5%] vs 115 125 [14.6%]; difference, 36.9%; 95% CI, 36.1%-37.7%; *P* < .001), and alcohol drinkers (16 382 [60.9%] vs 432 113 [51.3%]; difference, 9.6%; 95% CI, 8.9%-10.3%; *P* < .001) (Table 1). The age-standardized weighted prevalence of current e-cigarette use varied across US states and territories, from 1.0% in Puerto Rico to 6.2% in Guam (Figure). From 2016 to 2017, although the weighted prevalence of current e-cigarette use in several states slightly increased, more states had a decreased or unchanged prevalence of current e-cigarette use (eFigure in the Supplement).

**Table 1.** Participant Characteristics, Behavioral Risk Factor Surveillance System, 2016 to 2017

Characteristic	Participants, No. (%) (N = 894 997)^a^		*P* Value
	Electronic Cigarette Users (n = 28 907)	Electronic Cigarette Nonusers (n = 866 090)	
Age, y			
18-24	4856 (27.2)	45 291 (11.9)	<.001
25-34	5759 (25.3)	84 970 (16.9)	
35-44	4600 (17.6)	97 195 (16.1)	
45-54	5092 (14.3)	134 989 (16.9)	
55-64	5420 (11.1)	190 037 (17.0)	
≥65	3180 (4.6)	313 608 (21.2)	
Gender			
Male	15 068 (60.1)	375 940 (48.1)	<.001
Female	13 824 (39.8)	489 864 (51.9)	
Missing	15 (0.06)	286 (0.03)	
Race/ethnicity			
Non-Hispanic white	22 174 (70.6)	657 269 (62.2)	<.001
Non-Hispanic black	1991 (10.8)	69 739 (16.5)	
Hispanic	1650 (8.4)	68 173 (11.5)	
Other	3092 (10.2)	70 909 (9.8)	
Education			
Less than high school	2792 (14.3)	63 515 (13.4)	<.001
High school	10 317 (35.9)	235 746 (27.5)	
Attended college	10 093 (36.9)	237 429 (30.9)	
Graduated from college	5644 (12.8)	326 735 (27.9)	
Missing	61 (0.2)	2689 (0.3)	
Annual family income, $US			
<15 000	3762 (11.1)	72 639 (9.2)	<.001
15 000 to <25 000	5439 (17.2)	120 362 (14.2)	
25 000 to <35 000	3017 (9.9)	78 091 (8.7)	
35 000 to <50 000	3648 (12.1)	104 678 (11.3)	
≥50 000	9153 (35.1)	355 971 (41.3)	
Missing	3888 (14.7)	134 349 (15.3)	
Smoking status			
Current			<.001
Every day	10 752 (32.6)	82 186 (10.0)	
Some days	5230 (18.9)	32 939 (4.6)	
Former	8874 (28.6)	245 815 (23.8)	
Never	3908 (19.4)	500 331 (61.2)	
Missing	143 (0.6)	4819 (0.5)	
Alcohol intake^b^			
Nondrinker	11 644 (35.7)	413 935 (46.1)	<.001
Moderate drinking	13 184 (49.5)	383 863 (45.4)	
Heavy drinking	3198 (11.4)	48 250 (5.9)	
Missing	881 (3.4)	20 042 (2.7)	
Physical activity^c^			
Yes	20 058 (72.9)	627 170 (72.8)	.98
No	8310 (24.9)	224 415 (25.0)	
Missing	539 (2.1)	14 505 (2.1)	

^a^ Values are weighted.

^b^ Nondrinkers did not drink during the past 30 days. Moderate drinkers had 1 to 14 drinks per week for men and 1 to 7 drinks per week for women. Heavy drinkers had more than 14 drinks per week for men and more than 7 drinks per week for women.

^c^ Physical activity was defined as whether participants participate in any physical activities or exercises during the past month.

**Figure.**
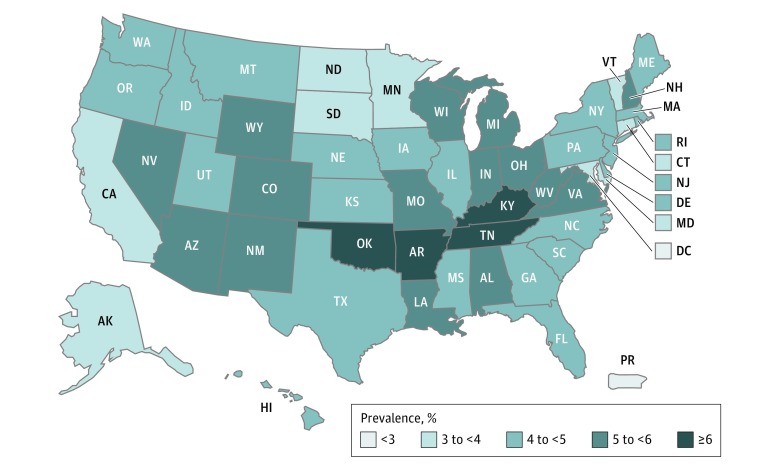
Age-Standardized Weighted Prevalence of Current Electronic Cigarette Use Among US Adults, Behavioral Risk Factor Surveillance System, 2016 to 2017 Prevalence estimates were weighted. The prevalence in Guam was 6.2% (not shown).

Across the types of state laws pertaining to e-cigarette use, the most commonly implemented laws were those prohibiting self-service displays of e-cigarettes, followed by those requiring a retail license to sell e-cigarettes and those banning the use of e-cigarettes and conventional cigarettes in restaurants, bars, and workplaces. As of January 1, 2017, only 7 states (District of Columbia, Kansas, Louisiana, Minnesota, North Carolina, Pennsylvania, and West Virginia) applied an excise tax to e-cigarettes, and only 3 states (California, District of Columbia, Hawaii) set 21 years as the minimum age to purchase e-cigarettes (Table 2). For each subtype of state laws regarding e-cigarettes, there was an increase in the number of states that implemented new state laws from 2016 to 2017.

**Table 2.** Proportion of States With Laws Regarding Electronic Cigarette Use and Proportion of Participants Exposed to Those Laws^a^

Laws	2016^b^			2017^c^		
	States	Proportion, No./Total No.		States	Proportion, No./Total No.	
		States^d^	Participants^e^		States^d^	Participants^e^
Prohibiting electronic cigarette use in indoor areas of private workplaces, restaurants, and bars	Delaware, Hawaii, New Jersey, North Dakota, Oregon, Puerto Rico, Utah, Vermont	8/53	49 850/465 627	California, District of Columbia, Delaware, Hawaii, New Jersey, North Dakota, Oregon, Puerto Rico, Utah, Vermont	10/53	67 422/429 370
Requiring retailer to purchase a license to sell electronic cigarettes	Arkansas, District of Columbia, Indiana, Iowa, Kansas, Louisiana, Minnesota, Montana, Rhode Island, Utah, Vermont	11/53	96 217/465 627	California, Arkansas, Connecticut, District of Columbia, Indiana, Iowa, Kansas, Louisiana, Minnesota, Montana, Rhode Island, Utah, Vermont, Pennsylvania, Washington	15/53	135 878/429 370
Prohibiting self-service displays of electronic cigarettes	Arkansas, Delaware, Florida, Hawaii, Idaho, Illinois, Indiana, Iowa, Kansas, Louisiana, Massachusetts, Minnesota, Nebraska, New York, New Mexico, North Dakota, Oklahoma, Oregon, South Dakota, Texas, Utah, Wyoming	22/53	222 429/465 627	Arkansas, California, Delaware, Florida, Hawaii, Idaho, Illinois, Indiana, Iowa, Kansas, Louisiana, Massachusetts, Maine, Minnesota, Nebraska, New York, New Mexico, North Dakota, Oklahoma, Oregon, South Dakota, Texas, Utah, Washington, Wyoming, Vermont	26/53	236 281/429 370
Prohibiting sales of tobacco products, including electronic cigarettes, to persons aged <21 y	Hawaii	1/53	7770/465 627	California, District of Columbia, Hawaii	3/53	19 811/429 370
Electronic cigarette tax	District of Columbia, Louisiana, Minnesota, North Carolina	4/53	35361/465 627	District of Columbia, Kansas, Louisiana, Minnesota, North Carolina, Pennsylvania, West Virginia	7/53	65 680/429 370

^a^ The term “states” includes all 50 US states, the District of Columbia, Puerto Rico, and Guam.

^b^ The state implemented the law no later than January 1, 2016.

^c^ The state implemented the law no later than January 1, 2017.

^d^ The proportion of states with the specific law.

^e^ The proportion of participants in the Behavioral Risk Factor Surveillance System 2016 to 2017 who resided in the states with the specific law.

We observed associations of several state laws regarding e-cigarettes with e-cigarette use. After adjustment for age, gender, race/ethnicity, education, family income, smoking status, alcohol intake, and physical activity, the odds ratios of current e-cigarette use associated with state-level regulations and policies were 0.90 (95% CI, 0.83-0.98) for state laws prohibiting e-cigarette use in indoor areas of private workplaces, restaurants, and bars; 0.90 (95% CI, 0.85-0.95) for state laws requiring retailers to purchase a license to sell e-cigarettes; 1.04 (95% CI, 0.99-1.09) for state laws prohibiting self-service displays of e-cigarettes; 0.86 (95% CI, 0.74-0.99) for state laws prohibiting sales of tobacco products, including e-cigarettes, to persons younger than 21 years; and 0.89 (95% CI, 0.83-0.96) for state laws applying taxes to e-cigarettes (Table 3).

**Table 3.** Association of State Laws Regarding Electronic Cigarettes With Current Electronic Cigarette Use, Behavioral Risk Factor Surveillance System, 2016 to 2017

State Laws	Implemented the Law, OR (95% CI)	
	No	Yes
Prohibiting electronic cigarette use in indoor areas of private workplaces, restaurants, and bars		
Model 1^a^	1 [Reference]	0.72 (0.67-0.78)
Model 2^b^	1 [Reference]	0.90 (0.83-0.98)
Requiring retailers to purchase a license to sell electronic cigarettes		
Model 1^a^	1 [Reference]	0.91 (0.87-0.96)
Model 2^b^	1 [Reference]	0.90 (0.85-0.95)
Prohibiting self-service displays of electronic cigarettes		
Model 1^a^	1 [Reference]	0.95 (0.91-0.99)
Model 2^b^	1 [Reference]	1.04 (0.99-1.09)
Prohibiting sales of tobacco products, including electronic cigarettes, to persons aged <21 y		
Model 1^a^	1 [Reference]	0.65 (0.57-0.75)
Model 2^b^	1 [Reference]	0.86 (0.74-0.99)
Electronic cigarette tax		
Model 1^a^	1 [Reference]	1.02 (0.95-1.10)
Model 2^b^	1 [Reference]	0.89 (0.83-0.96)

Abbreviation: OR, odds ratio.

^a^ Multivariable model 1 was adjusted for age (years) and gender.

^b^ Multivariable model 2 included multivariable model 1 plus race/ethnicity, education, family income, smoking status, alcohol intake, and physical activity.

In stratified analyses, there was an association between state laws regarding e-cigarettes and e-cigarette use among adults aged 45 to 64 years, female participants, nonwhite participants, those with lower education levels, and those with lower family income (eTable in the Supplement). For example, the odds ratios of current e-cigarette use associated with state laws prohibiting e-cigarette use in indoor areas of private workplaces, restaurants, and bars were 0.95 (95% CI, 0.81-1.12) for adults aged 18 to 24 years, 1.00 (95% CI, 0.88-1.13) for adults aged 25 to 44 years, 0.68 (95% CI, 0.59-0.79) for adults aged 45 to 64 years, and 0.82 (95% CI, 0.62-1.10) for adults aged 65 years or older (eTable in the Supplement). However, for e-cigarette taxes, there was an association with e-cigarette use among adults aged 18 to 24 years and those 65 years and older. The odds ratios of current e-cigarette use associated with state laws applying taxes to e-cigarettes were 0.81 (95% CI, 0.68-0.97) for adults aged 18 to 24 years, 0.93 (95% CI, 0.83-1.04) for adults aged 25 to 44 years, 0.96 (95% CI, 0.85-1.09) for adults aged 45 to 64 years, and 0.78 (95% CI, 0.62-0.98) for adults aged 65 years or older (eTable in the Supplement).

## Discussion

In a large, nationwide, state-based heath survey of US adults, we found associations of e-cigarette use with state laws that prohibit e-cigarette use in indoor areas of private workplaces, restaurants, and bars; that require a retail license to sell e-cigarettes; that prohibit sales of tobacco products, including e-cigarettes, to persons younger than 21 years; and that apply taxes to e-cigarettes. To our knowledge, this is the first study estimating the potential outcomes of state laws on e-cigarette use among US adults. Consistent with previous reports,^4,34,35^ our findings show that the age-standardized prevalence of current e-cigarette use varied across states. The reasons for the state variations remain to be elucidated, but these findings provide clues about the potential outcomes associated with e-cigarette laws. At the state level, a CDC report^33^ documented that the legislative activity regarding e-cigarettes was initiated in 2010, increased between 2013 and 2015, and peaked in 2015. One recent study^7^ found a substantial decrease in current e-cigarette use among US adults between 2014 and 2016, which may indicate a positive outcome of state laws to curb the increasing trend of e-cigarette use.

Taxation and smoke-free laws (ie, prohibiting cigarette smoking in restaurants, bars, and workplaces) have been proven to help increase smoking cessation and reduce the prevalence of conventional cigarette use in the US general population.^39,40,41^ It is interesting and reassuring that e-cigarette–related regulations similar to the aforementioned laws may also help reduce e-cigarette use. We also observed an association of retail license requirements with a lower rate of e-cigarette use in adults. The retail license requirement as a strategy to regulate e-cigarettes has aroused public attention, because a recent study^42^ found that youth who live in areas with strong tobacco vendor licensing requirements have lower rates of tobacco use and e-cigarette use. However, federal law does not require retailers to have a license to sell e-cigarettes,^31^ and fewer than one-third of states require e-cigarette retailers to be licensed. Before January 1, 2017, only Hawaii, California, and the District of Columbia implemented laws prohibiting sales of e-cigarettes to persons younger than 21 years (also known as “tobacco 21 laws”).^32^ Therefore, further studies are needed to evaluate the association between implementation of the tobacco 21 laws and e-cigarette use among adolescents and young adults in the United States.

Our study has public health implications. e-Cigarette use has emerged as a major public health issue. Millions of adults and youth in the United States are using e-cigarettes.^5^ e-Cigarettes may aid in smoking cessation for some individuals, but they may also cause harm to others.^1^ At the time e-cigarettes entered the US market, they were commonly regarded as a safer substitute for conventional cigarettes, and many smokers initiated e-cigarette use with an intention to quit smoking.^3^ Our results show that more than one-half (51.5%) of current e-cigarette users are also current conventional cigarette smokers. Despite their popularity, e-cigarettes have not been approved by the US Food and Drug Administration for smoking cessation, and the efficacy and safety of e-cigarettes compared with US Food and Drug Administration–approved cessation aids warrant further investigation.^15,16,43^

Moreover, e-cigarette use might be associated with youth or never smokers transitioning to combustible tobacco products.^17,44,45,46^ Whether e-cigarette use is a possible gateway to the use of combustible cigarettes and other substances (eg, alcohol or illegal drugs) remains to be determined.^46,47,48,49^ Widespread use of e-cigarettes can also lead to secondhand exposure to nicotine and other toxic substances from e-cigarettes.^50,51,52^ Therefore, careful consideration of the potential benefits and harms of e-cigarettes is needed in determining the net public health consequences associated with use of e-cigarettes.

In addition to e-cigarette regulations at the federal level by the US Food and Drug Administration, state and local regulations and policies regarding e-cigarettes can help reduce the public health risks associated with e-cigarettes, particularly among at-risk populations.^33^ Recent studies^8,53^ have reported an emerging increase in the prevalence of current e-cigarette use among US youth and young adults aged 18 to 24 years. This increase is concerning because nicotine exposure from e-cigarettes may harm brain development in young people.^54^ Our stratified analyses showed that among adults aged 18 to 24 years, e-cigarette taxes were associated with lower rates of e-cigarette use. Although the reasons for this association remain unclear and warrant further investigation, it indicates that state-level regulations may be a hopeful approach to curb the increase in e-cigarette use among young adults.

### Strengths and Limitations

The major strength of our study is the use of data from a nationwide, state-based, large-scale health survey of US adults. Because of the special considerations for both nationwide and state-level sampling during survey design, BRFSS is uniquely positioned to address state variations and determinants of health behaviors, including e-cigarette use. There are also several limitations to this study. First, the information on e-cigarette use and other lifestyle factors (eg, cigarette smoking and alcohol drinking) were collected via self-reports, which may be subject to misreporting or recall bias. Second, we did not have information on the brands and subtypes of e-cigarettes and types of e-cigarette liquid. Whether the state laws would have differential associations with the different subtypes of e-cigarettes warrants further investigation. Third, participants were regarded as being exposed to the e-cigarette–related state laws in the specific state where they resided at the time of the BRFSS survey. It is possible that some participants moved or worked between states, which may have resulted in misclassification of the exposure to state laws. Fourth, although we have adjusted for a variety of covariates related to both state laws and e-cigarette use, the BRFSS did not collect detailed information on the years that participants resided in the same states and participants’ knowledge of the state laws regarding e-cigarette. Therefore, residual confounding may still exist.

## Conclusions

Findings from this study suggest that US state regulations regarding e-cigarettes may be associated with reduced e-cigarette use among US adults. Because of the dynamic nature of state law legislation and implementation, future studies are needed to continue monitoring the patterns of state-level variations in e-cigarette use and the outcomes of changing state laws on e-cigarette use.

## Notes

Supplement. **eFigure. **Change in Age-Standardized Prevalence of Current Electronic Cigarette Use Among US Adults From 2016 to 2017 in the Behavioral Risk Factor Surveillance System **eTable. **Stratified Analysis for the Associations of State Laws Regarding Electronic Cigarettes With Current Electronic Cigarette Use, Behavioral Risk Factor Surveillance System 2016-2017
