# Supplementary material for: Association of Electronic Cigarette Regulations With Electronic Cigarette Use Among Adults in the United States
Source: JAMA Netw Open. 2020 Jan 31;3(1):e1920255. doi: 10.1001/jamanetworkopen.2019.20255 (PMC7042861; doi:10.1001/jamanetworkopen.2019.20255)
Supplement: Supplement. — eFigure. Change in Age-Standardized Prevalence of Current Electronic Cigarette Use Among US Adults From 2016 to 2017 in the Behavioral Risk Factor Surveillance System eTable. Stratified Analysis for the Associations of State Laws Regarding Electronic Cigarettes With Current Electronic Cigarette Use, Behavioral Risk Factor Surveillance System 2016-2017 [file jamanetwopen-3-e1920255-s001.pdf]

Supplementary Online Content

Du Y, Liu B, Xu G, et al. Association of electronic cigarette regulations with electronic cigarette use among adults in the United States. *JAMA Netw Open*. 2020;3(1):e1920255. doi:10.1001/jamanetworkopen.2019.20255

eFigure. Change in Age-Standardized Prevalence of Current Electronic Cigarette Use Among US Adults From 2016 to 2017 in the Behavioral Risk Factor Surveillance System

eTable. Stratified Analysis for the Associations of State Laws Regarding Electronic Cigarettes With Current Electronic Cigarette Use, Behavioral Risk Factor Surveillance System 2016-2017

This supplementary material has been provided by the authors to give readers additional information about their work.

© 2020 Du Y et al. *JAMA Network Open.*

eFigure. Change in Age-Standardized Prevalence of Current Electronic Cigarette Use Among US Adults From 2016 to 2017 in the Behavioral Risk Factor Surveillance System

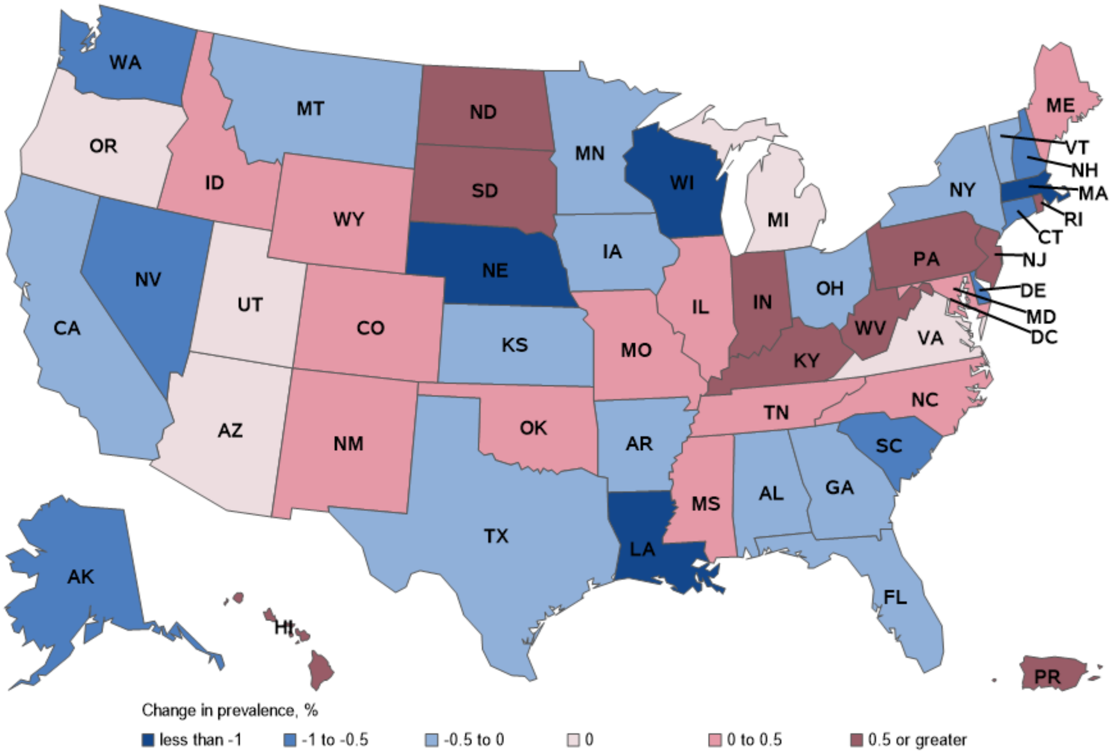

Prevalence estimates were weighted.

© 2020 Du Y et al. *JAMA Network Open.*

eTable. Stratified Analysis for the Associations of State Laws Regarding Electronic Cigarettes With Current Electronic Cigarette Use, Behavioral Risk Factor Surveillance System 2016-2017

**Variables**	**Odds Ratio (95% CI)**		**P value for interaction**
	**Not implemented the law**	**Implemented the law**	
**Prohibiting e-cigarette use in indoor areas of private workplaces, restaurants, and bars**			
Age			
18-24	1.00 (reference)	0.95 (0.81-1.12)	<.001
25-44	1.00 (reference)	1.00 (0.88-1.13)	
45-64	1.00 (reference)	0.68 (0.59-0.79)	
65+	1.00 (reference)	0.82 (0.62-1.10)	
Gender			
Male	1.00 (reference)	0.93 (0.84-1.04)	.001
Female	1.00 (reference)	0.85 (0.75-0.96)	
Race/Ethnicity			
White	1.00 (reference)	1.01 (0.92-1.11)	<.001
Non-white	1.00 (reference)	0.76 (0.66-0.88)	
Education			
High school or less	1.00 (reference)	0.86 (0.76-0.96)	.03
More than high school	1.00 (reference)	0.93 (0.84-1.04)	
Family income			
<$50,000	1.00 (reference)	0.82 (0.74-0.92)	.03
$50,000 or more	1.00 (reference)	1.01 (0.88-1.16)	
Smoking status			
Current smoker (every day)	1.00 (reference)	0.98 (0.85-1.13)	.002
Current smoker (some days)	1.00 (reference)	0.81 (0.66-0.98)	
Former smoker	1.00 (reference)	0.92 (0.80-1.06)	
Never smoker	1.00 (reference)	0.88 (0.73-1.05)	
**Requiring retailer to purchase a license to sell e-cigarettes**			

© 2020 Du Y et al. *JAMA Network Open.*

Age			
18-24	1.00 (reference)	0.90 (0.80-1.01)	<.001
25-44	1.00 (reference)	0.95 (0.87-1.04)	
45-64	1.00 (reference)	0.85 (0.77-0.93)	
65+	1.00 (reference)	0.88 (0.75-1.03)	
Gender			
Male	1.00 (reference)	0.91 (0.85-0.98)	<.001
Female	1.00 (reference)	0.89 (0.82-0.96)	
Race/Ethnicity			
White	1.00 (reference)	0.95 (0.89-1.01)	.02
Non-white	1.00 (reference)	0.81 (0.72-0.93)	
Education			
High school or less	1.00 (reference)	0.86 (0.80-0.93)	.009
More than high school	1.00 (reference)	0.95 (0.88-1.03)	
Family income			
<$50,000	1.00 (reference)	0.87 (0.81-0.94)	.01
$50,000 or more	1.00 (reference)	0.96 (0.87-1.05)	
Smoking status			
Current smoker (every day)	1.00 (reference)	0.90 (0.83-0.99)	.008
Current smoker (some days)	1.00 (reference)	0.83 (0.72-0.95)	
Former smoker	1.00 (reference)	0.97 (0.89-1.07)	
Never smoker	1.00 (reference)	0.89 (0.78-1.02)	
**Prohibiting self-service displays of e-cigarettes**			
Age			
18-24	1.00 (reference)	1.00 (0.90-1.11)	.08
25-44	1.00 (reference)	1.01 (0.94-1.09)	
45-64	1.00 (reference)	1.12 (1.03-1.22)	
65+	1.00 (reference)	1.08 (0.94-1.25)	
Gender			
Male	1.00 (reference)	1.07 (1.00-1.14)	.23
Female	1.00 (reference)	1.00 (0.93-1.07)	

© 2020 Du Y et al. *JAMA Network Open.*

Race/Ethnicity			
White	1.00 (reference)	1.02 (0.97-1.08)	.23
Non-white	1.00 (reference)	1.09 (0.98-1.20)	
Education			
High school or less	1.00 (reference)	1.07 (0.99-1.14)	.14
More than high school	1.00 (reference)	1.01 (0.94-1.08)	
Family income			
<$50,000	1.00 (reference)	1.07 (1.00-1.14)	.98
$50,000 or more	1.00 (reference)	1.02 (0.94-1.11)	
Smoking status			
Current smoker (every day)	1.00 (reference)	1.07 (0.98-1.15)	.65
Current smoker (some days)	1.00 (reference)	1.00 (0.90-1.12)	
Former smoker	1.00 (reference)	1.11 (1.02-1.21)	
Never smoker	1.00 (reference)	0.93 (0.83-1.04)	
**Prohibiting sales of tobacco products, including e-cigarettes, to persons aged <21 years**			
Age			
18-24	1.00 (reference)	0.80 (0.59-1.07)	.001
25-44	1.00 (reference)	1.09 (0.89-1.34)	
45-64	1.00 (reference)	0.52 (0.38-0.71)	
65+	1.00 (reference)	0.73 (0.43-1.24)	
Gender			
Male	1.00 (reference)	0.90 (0.75-1.08)	.02
Female	1.00 (reference)	0.77 (0.60-0.99)	
Race/Ethnicity			
White	1.00 (reference)	1.03 (0.85-1.26)	.005
Non-white	1.00 (reference)	0.71 (0.57-0.88)	
Education			
High school or less	1.00 (reference)	0.71 (0.56-0.89)	.39
More than high school	1.00 (reference)	0.99 (0.82-1.20)	
Family income			
<$50,000	1.00 (reference)	0.74 (0.60-0.91)	.12

© 2020 Du Y et al. *JAMA Network Open*.

$50,000 or more	1.00 (reference)	1.00 (0.80-1.25)	
Smoking status			
Current smoker (every day)	1.00 (reference)	0.75 (0.56-1.02)	.10
Current smoker (some days)	1.00 (reference)	0.77 (0.54-1.10)	
Former smoker	1.00 (reference)	1.04 (0.83-1.30)	
Never smoker	1.00 (reference)	0.79 (0.58-1.08)	
**E-cigarette tax**			
Age			
18-24	1.00 (reference)	0.81 (0.68-0.97)	.02
25-44	1.00 (reference)	0.93 (0.83-1.04)	
45-64	1.00 (reference)	0.96 (0.85-1.09)	
65+	1.00 (reference)	0.78 (0.62-0.98)	
Gender			
Male	1.00 (reference)	0.89 (0.80-0.98)	.02
Female	1.00 (reference)	0.91 (0.81-1.01)	
Race/Ethnicity			
White	1.00 (reference)	0.89 (0.82-0.97)	.30
Non-white	1.00 (reference)	0.91 (0.77-1.08)	
Education			
High school or less	1.00 (reference)	0.89 (0.83-0.96)	.01
More than high school	1.00 (reference)	0.92 (0.83-1.02)	
Family income			
<$50,000	1.00 (reference)	0.90 (0.81-0.99)	.007
$50,000 or more	1.00 (reference)	0.94 (0.82-1.07)	
Smoking status			
Current smoker (every day)	1.00 (reference)	0.87 (0.77-0.99)	.06
Current smoker (some days)	1.00 (reference)	0.92 (0.77-1.10)	
Former smoker	1.00 (reference)	0.91 (0.80-1.04)	
Never smoker	1.00 (reference)	0.91 (0.75-1.09)	

Adjusted for age, sex, race/ethnicity, education, family income, smoking status, alcohol intake, and physical activity.

© 2020 Du Y et al. *JAMA Network Open*.
